# Development of pH-Dependent Magnetically Actuated Millirobot for Colon-Targeted Delivery of Diverse Drug Types

**DOI:** 10.3390/mi17050610

**Published:** 2026-05-15

**Authors:** Xiaoyu Li, Weibin Rong, Lefeng Wang, Hongda Jia, Xianghe Meng, Hui Xie

**Affiliations:** State Key Laboratory of Robotics and System, Harbin Institute of Technology, Harbin 150080, China

**Keywords:** magnetically driven millirobot, oral administration, colon drug delivery systems, pH-dependent film

## Abstract

Oral administration is an ideal route for colon-targeted drug delivery; however, precise delivery to the colon remains a challenge. This work presents a magnetically actuated millirobot combined with a traditional pH-dependent strategy. It aims to combine the advantages of the two methods: under normal physiological conditions, it enables autonomous targeted drug delivery, effectively reducing manipulation costs; in abnormal physiological environments, precise targeted delivery can be achieved via external magnetic intervention. The millirobot uses a magnetic composite shell and a pH-dependent film to encapsulate drug carriers. The pH-dependent film ensures an appropriate delay in drug release under different simulated pH conditions. The magnetic composite shell exhibits satisfactory magnetic responsiveness and can perform stable tumbling motion on the surface of the ex vivo intestinal tract, demonstrating good controllability and motility. Furthermore, the millirobot can carry different types of drug carriers to achieve tunable drug-release rates, thereby improving its versatility. These experimental results demonstrate that this pH-dependent magnetically actuated millirobot is a promising platform for reducing manipulation costs and enhancing the reliability of colon-targeted drug delivery.

## 1. Introduction

The colon is susceptible to diseases such as ulcerative colitis, Crohn’s disease, and intestinal cancer [1]. Achieving precise delivery of drugs to the colonic target area can effectively prevent nonspecific absorption in the small intestine, increase local drug concentration, and reduce systemic dosage and side effects [2]. At the same time, the low protease activity in the colon makes it an ideal site for the absorption of protein- and peptide-based drugs [3]. Compared with intravenous injection, rectal administration, and other methods, oral administration offers significant advantages in convenience and non-invasiveness and is the optimal administration route for colon-specific delivery [4]. However, the complex physiological environment of the gastrointestinal tract (GIT) poses a severe challenge to oral colon-targeted drug delivery [5].

To address the above challenges, traditional colon-targeting strategies are used, namely colon-specific drug delivery systems (CDDS). They utilize the unique physiological characteristics of the colon as triggers for drug release without employing other triggering methods [6]. Various strategies have been extensively studied. Among them, the traditional pH-dependent strategy employs pH-dependent polymers as coating materials or carrier matrices based on the pH gradient along the GIT. These polymers remain stable in acidic environments but swell or dissolve in neutral environments, thereby releasing the drug [7]. Although phased progress has been made in this field, the drug release effect of such delivery systems is still significantly affected by the physiological conditions of the GIT, for instance, in the disease state, the gastrointestinal transit time and pH value may change abnormally, while microbiota disorders will weaken the release efficiency of enzyme-triggered delivery systems, all of which are likely to lead to the failure of targeted delivery [8,9]. To make up for the above limitations, researchers have undertaken a series of optimization efforts, primarily by combining specific drug-delivery methods [10,11,12,13]. These optimization strategies mainly reduce the interference of physiological factors in the drug release process by introducing multiple triggering conditions, thereby improving triggering stability to a certain extent. However, there may still be problems of triggering failure, and the multi-trigger method may increase the structural complexity of the drug delivery device [14].

A magnetically driven miniature robot refers to a small-scale robot that is remotely and wirelessly actuated and manipulated by an external magnetic field, with a typical size ranging from micrometer to millimeter scale [15]. At present, there are four main types of external energy actuation methods, namely magnetic actuation, acoustic actuation, optical actuation, and electrical actuation [16]. Limited by light absorption and scattering in biological tissues, as well as the charge dissipation of electric fields in conductive physiological environments, optical and electrical actuation show weak tissue penetration performance. Both methods are therefore more applicable to in vitro experiments or superficial tissue regions [17,18]. Acoustic actuation has favorable tissue penetration and biocompatibility, yet it is limited in terms of motion control precision [19,20]. Magnetic fields suffer from obvious attenuation with increasing distance, which means high magnetic field strength is required for deep tissue actuation (especially gradient magnetic fields), and its application is restricted for patients with internal metallic implants, especially under high-intensity magnetic fields. Nevertheless, compared with other actuation methods, magnetic actuation has prominent strengths such as remote precise manipulation, superior tissue penetration, and excellent biocompatibility [21]. With these advantages, magnetically driven miniature robots provide a novel approach for colon-targeted drug delivery [22].

In the field of intestinal drug delivery, magnetically driven miniature robots are typically designed with a biocompatible shell to load magnetic materials and drugs [23], or with a magnetic composite shell to carry drugs [24]. Rotating magnetic fields and gradient magnetic fields are the primary driving magnetic fields, which generate magnetic torque and magnetic force respectively to actuate robot motion. The gradient magnetic field drives the robot directly through magnetic dragging, where the shape does not affect its propulsion principle but mainly influences friction and safety, such as capsule-shaped or spherical designs [25]. The rotating magnetic field uses magnetic torque to drive the robot to follow the rotation, requiring structural design to convert the rotational motion into translational motion. The tumbling/rolling motion of robots relies on the friction force generated between the robot and the intestinal surface during rotation, such as sheet-like [26], spherical [27], cylindrical [28], or capsule-like robots with helical ribs [29]. Additionally, using a rotating magnetic field to drive the robot to generate fluid reaction forces [30] or to form a “self-magnetic field” [31] to propel the robot forward is also a feasible approach. Their drug-release methods are primarily classified as active-controlled release [32] and passively triggered release [33]. The key difference is that active release can be triggered by external signals that induce changes in the robot, such as the actuation of mechanical structures or the magnetothermal effect, leading to drug release. In contrast, passive release relies solely on the internal physiological environment (e.g., pH, enzymes) to cause physical or chemical changes in the delivery system. The movement trajectory and speed of robots can be flexibly regulated by an external magnetic field, enabling them to adapt to the complex physiological environment of the GIT and holding promise for achieving precise drug delivery.

Current studies have combined pH-dependent strategies with miniature robot platforms. In most cases, pH-dependent materials are merely utilized to prevent premature drug leakage during locomotion, as exemplified by pH-responsive parent–child miniature robots [34] and self-propelled miniature robots [35]. Particularly for existing designs of magnetically actuated pH-responsive hybrid miniature robots, continuous robotic locomotion must be maintained throughout the drug delivery process. In addition, the motion control range of magnetically driven miniature robots is strictly limited by the working space of external magnetic control equipment, and high-precision magnetic control devices are structurally complex. Together, these two factors result in a bulky overall system. Therefore, such drug delivery carriers relying on uninterrupted magnetic actuation present obvious limitations: their movement and drug release processes require real-time external intervention, which increases the manipulation cost of miniature robots and compromises the convenience of oral administration.

Compared with other sites, such as the brain and blood vessels, traditional colon-targeted delivery technologies are relatively well established. If magnetic drive technology can be organically combined with these conventional strategies, it is anticipated that this integration will yield complementary advantages and construct a new type of colon-targeted delivery system that is both reliable and convenient. Specifically, when the physiological conditions of the GIT are normal, the drug is delivered by traditional targeting methods without requiring full-course magnetic field intervention, thereby reducing treatment costs. In cases where gastrointestinal conditions are abnormal, the carrier can be precisely manipulated via an external magnetic field to reach the optimal release site in the colon, ensuring effective drug delivery.

Based on this concept, we have developed a miniature robot that combines a traditional pH-dependent strategy with magnetic drive technology. The robot is of millimeter scale (a millirobot), with an outer diameter of 8 mm and a height of 2.6 mm. The magnetic composite shell of the millirobot is designed as a tablet-like structure with smooth rounded edges, mimicking conventional oral tablets. Such tablet-shaped configurations are widely used in oral drug delivery due to their well-established safety and non-irritating properties. Combined with the soft, biocompatible Polydimethylsiloxane (PDMS) matrix, this design is expected to help reduce mechanical irritation and improve safety during intestinal locomotion. NdFeB magnetic powder is mixed inside the shell to endow it with strong magnetism. At a magnetic flux density of 8 mT, a uniform rotating magnetic field can drive the millirobot to tumble on the surface of the ex vivo intestinal tract, and its maximum controllable speeds in air and liquid environments can reach approximately 8 cm/s and 10 cm/s, respectively. One side of the magnetic shell of the millirobot is covered with an Eudragit L100 film of a specific thickness as a pH-dependent film. In vitro gastrointestinal simulation experiments show that a drug-release delay of approximately 2.5–3 h can be achieved in a simulated small-intestinal pH environment. Under abnormal pH conditions, in vitro experiments simulating high- and low-pH environments indicate that the Eudragit L100 film covered on the surface of the millirobot exhibits appropriate dissolution properties. Appropriate dissolution properties of the pH-dependent film and a high movement speed can ensure that the millirobot can be controlled in vitro under abnormal pH conditions. Meanwhile, the millirobot can carry three different types of drug carriers, namely liquid, semi-solid, and solid, and exhibit three drug release rates (fast, medium, and slow), which indicates that the millirobot has the potential to adapt to the treatment needs of different intestinal diseases.

The results from in vitro drug release experiments and ex vivo locomotion performance tests on porcine intestinal tracts confirm that the proposed magnetically driven pH-dependent millirobot effectively integrates the passive reliability of traditional targeting strategies with the active controllability of magnetic actuation. Traditional targeting strategies enable autonomous, trigger-based release under normal physiological conditions without requiring additional manipulation, while magnetic actuation serves as a backup insurance mechanism for the pH-dependent system under abnormal physiological conditions, thereby ensuring the reliability of drug delivery. Meanwhile, it can load various drug carriers to address diverse drug-release scenarios. In summary, this study is expected to provide a versatile platform for colon-specific drug delivery.

## 2. Materials and Methods

### 2.1. Materials

Polyethylene glycol diacrylate (PEGDA, MW = 675), methylene blue, ethyl cellulose (EC), and triethyl citrate were purchased from Shanghai Aladdin Biochemical Technology Co., Ltd., Shanghai, China. Sodium chloride (NaCl) was acquired from Sinopharm Chemical Reagent Co., Ltd., Beijing, China. Photoinitiator 1173 was obtained from Shanghai Yinchang Co., Ltd., Shanghai, China. Polydimethylsiloxane (PDMS, DC184) was purchased from Dow Corning Corporation, Midland, MI, USA. Eudragit L100 and S100 were kindly provided as samples by Shanghai Changwei Pharmaceutical Excipients Technology Co., Ltd., Shanghai, China. Additionally, 0.01 mol/L phosphate-buffered solution (PBS, pH = 7.2) was purchased from Biosharp, Hefei, China. Neodymium-iron-boron magnetic powder (NdFeB, model MQFP) was obtained from Magnequench Corporation, Pendleton, IN, USA. Standard hydrochloric acid and sodium hydroxide solutions were purchased from Shenzhen Bolinda Technology Co., Ltd., Shenzhen, China. Carbopol 974P was provided as a sample by Lubrizol Corporation, Wickliffe, OH, USA.

### 2.2. Fabrication of Millirobots

**Figure 1:**
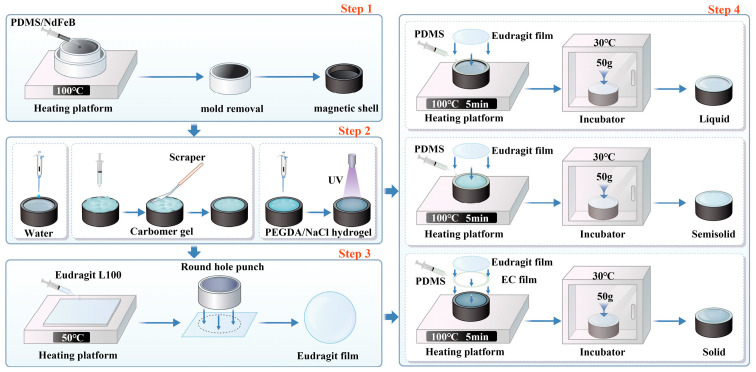
Preparation steps of the millirobot, steps 1–4 correspond to Section 2.2.1, Section 2.2.2, Section 2.2.3 and Section 2.2.4, respectively.

As shown in Figure 1, the millirobot was fabricated through a four-step process: first, the magnetic composite shell was fabricated (Figure 1 Step 1); second, the drug was loaded (Figure 1 Step 2); third, a pH-dependent Eudragit film was fabricated (Figure 1 Step 3); and finally, the structure was sealed (Figure 1 Step 4).

#### 2.2.1. Fabrication of the Millirobot Shell

The millirobot shell was fabricated from a mixture of PDMS and NdFeB magnetic powder. A micro-nano 3D printer (Micro P215 plus, Jiangmen Xingsheng Precision Manufacturing Co., Ltd., Jiangmen, China) was used to print the mold, which was designed as a two-part structure consisting of a ring and a base to facilitate demolding. The PDMS matrix, curing agent, and NdFeB were mixed uniformly at a mass ratio of 10:1:10, and the mixture was then placed in a vacuum drying oven for 30 min to remove air bubbles. Approximately 180 mg of the mixture was injected into the mold, which was then placed on a constant-temperature stage at 100 °C for 1 h to cure. After cooling, the ring, together with the millirobot shell, was removed from the base as an integrated unit. The ring was then carefully separated from the shell to obtain the complete millirobot shell. The fabricated millirobot shell has an outer diameter (OD) of 8 mm and an inner diameter (ID) of 5.5 mm and is used for drug loading.

#### 2.2.2. Preparation of the Drug Carriers

In this experiment, methylene blue was used as the model dye, and deionized water, Carbopol gel, and PEGDA hydrogel were selected as three representative types of drug carriers to verify the carrier adaptability of the millirobot.

Aqueous solution: Methylene blue (0.4 wt%) was dissolved in deionized water, and acetic acid (2%) was added to adjust the pH to approximately 3.

Carbopol gel: Carbopol 974P (4 wt%) and methylene blue (0.4 wt%) were dissolved in deionized water and stirred for 3 h to obtain a homogeneous solution. The solution was degassed in a vacuum drying oven for 10 min, and its pH was adjusted to 5 using NaOH to form the drug-loaded gel.

PEGDA hydrogel: Photoinitiator 1173 (1 wt%) and methylene blue (0.4 wt%) were added to PEGDA, and the mixture was stirred for 3 h. NaCl (equal mass to PEGDA) was then added and stirred for 30 min. The NaCl particles, sieved to 30–74 μm using a 200-mesh sieve and a 30-μm nylon membrane, were used to create porous structures. The resulting PEGDA/NaCl solution was stored in the dark until use.

#### 2.2.3. Preparation of Eudragit Film

Anhydrous ethanol (32 g) and deionized water (4 g) were mixed, followed by the addition of Eudragit L100 (4 g) and triethyl citrate (0.8 g). The mixture was stirred in a constant-temperature water bath at 60 °C for 6 h to obtain a uniform solution, which was then placed in a vacuum drying oven and treated under vacuum for 10 min to remove air bubbles. A certain volume of Eudragit solution was poured onto a 5 × 5 cm^2^ polytetrafluoroethylene (PTFE) plate, which was then placed in a constant-temperature drying oven at 50 °C for 12 h to obtain the Eudragit film. After separating the film from the plate, an 8-mm-diameter circular punch was used to cut it. A micrometer was used to measure five points of the film to ensure that the thickness error of the cut film was within ±10 μm. The circular film was stored in a plastic Petri dish at room temperature until use.

#### 2.2.4. Sealing of the Millirobot with Eudragit Film

The sealing method varied depending on the type of drug carrier, with the core requirement of ensuring tight adhesion and unidirectional drug release.

For aqueous solution and Carbopol gel: For the drug-loaded aqueous solution, 20 μL was injected into the millirobot, and a needle was used to distribute the solution uniformly within it. For the drug-loaded Carbopol gel, after injecting the gel into the magnetic millirobot, a scraper was used to remove the gel that exceeded the surface of the robot. The cut circular film was sealed on the surface of the millirobot. Specifically, first, the PDMS matrix and curing agent were mixed uniformly at a ratio of 8:1 as an adhesive, then the adhesive was applied to the frame of the millirobot, which was then placed on a constant-temperature heating stage at 80 °C for 5 min, and then transferred to a constant-temperature oven at 30 °C for 12 h to ensure PDMS curing. During the curing process, a 50 g weight was placed on the film to ensure tight adhesion and keep it attached to the millirobot at all times.

For PEGDA/NaCl hydrogel: The PEGDA/NaCl solution (35 μL) was injected into a mold slightly smaller than the magnetic shell of the millirobot. The solution was then irradiated with UV light (150 mW/cm^2^) for 5 min to cure, forming a cylindrical hydrogel (5 mm diameter). The resulting hydrogel was removed from the mold and placed into the magnetic shell. Meanwhile, EC was dissolved in anhydrous ethanol at 10 wt% and stirred until completely dissolved. The solution was degassed under vacuum for 10 min to remove air bubbles. EC solution (approximately 10 μL) was cast onto an annular silicone rubber with an OD of 8 mm and an ID of 4 mm. The Eudragit film was gently pressed onto the EC solution, then cured on a constant-temperature drying stage at 60 °C for 30 min, after which the silicone rubber was removed to obtain a double-layer film. The double-layer film (with the EC film on the bottom) was then bonded to the magnetic shell using the same PDMS adhesive method described above for the aqueous solution and gel carriers.

**Figure 2:**
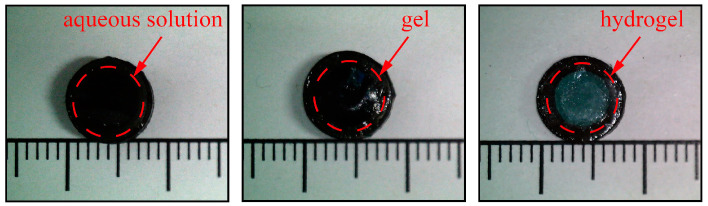
Photographs of the millirobots loaded with different drug carriers. From left to right: millirobots loaded with aqueous solution, gel, and hydrogel, respectively.

The fabricated millirobots are displayed in Figure 2.

### 2.3. Determination of Eudragit Film Mass Loss

The films with different mass ratios of Eudragit L100 and S100 were prepared as described in Section 2.2.3. Each film was bonded to a PDMS/NdFeB shell and weighed, with the initial weight recorded as *m*_1_. They were then placed in vials containing 50 mL of PBS, with pH ranging from 6 to 8 in increments of 0.5. The vials were placed in a constant-temperature water bath shaker at 37 °C and 100 rpm. After 2 h, the shell with the bonded film was taken out, and the film surface was gently blotted dry with absorbent paper. The weight was then recorded as *m*_2_. The mass loss Δ*m* of the Eudragit film was calculated using Equation (1). The composition of the Eudragit L100/S100 films is shown in Table 1.

(1) $$\Delta m=m_{1}-m_{2}$$

### 2.4. Determination of Hydrogel Properties

#### 2.4.1. Determination of Swelling Properties

Cylindrical PEGDA hydrogels with different NaCl contents (PEGDA: NaCl mass ratios of 1:0, 2:1, and 1:1) were prepared. The hydrogels had a diameter of 5 mm and a height of 2 mm, matching the internal hydrogel of the millirobot. The hydrogels were placed in a vacuum drying oven at 30 °C for 24 h, and the weight at this time was recorded as *m*_3_. They were then immersed in PBS solution at 37 °C. At predetermined time intervals, the hydrogels were removed, the water droplets on the surface of the hydrogels were removed, and the weight was recorded as *m*_4_. The swelling ratio *R*_1_ of the hydrogels could be calculated using Equation (2):

(2) $$R_{1}=\frac{m_{4}}{m_{3}}$$

After the hydrogels reached equilibrium swelling, their dimensions were measured using a micrometer, and the volume change was calculated. The volume swelling ratio *R*_2_ at this time was calculated:

(3) $$R_{2}=\frac{V_{1}-V_{0}}{V_{0}}$$

In the above equation, *V*_0_ represents the initial volume of the hydrogel, and *V*_1_ is the volume at equilibrium swelling. The volume of the hydrogel was calculated according to the standard cylinder formula.

#### 2.4.2. Fourier Transform Infrared Spectroscopy (FT-IR)

The chemical structures of the PEGDA hydrogel, NaCl powder, and PEGDA/NaCl hydrogel were characterized using a Fourier transform infrared spectrometer (Nicolet iS5, Thermo Fisher, Waltham, MA, USA) equipped with an attenuated total reflection (ATR) accessory. All spectra were recorded in ATR mode over the range of 4000–400 cm^−1^ at a resolution of 4 cm^−1^ with 32 scans per sample. Prior to measurement, each sample was placed directly on the ATR crystal, and spectra were collected under dry ambient conditions.

### 2.5. Determination of Millirobot Movement Speed

The movement speed of the millirobot was evaluated on the surface of the fresh ex vivo porcine colon under a rotating magnetic field (8 mT) with varying frequencies. The porcine colon was sliced and placed in a Petri dish, which was then placed inside the magnetic coil. The movement speeds were measured in air (0.5–4 Hz) and in water (1–9 Hz) environments. Five measurements were performed, each using a different porcine colon. For each frequency, the time *t* required for the millirobot to travel a fixed distance *d* was recorded using a video camera. The average speed *V* was calculated using Equation (4):

(4) $$V=\frac{d}{t}$$

Specifically, the speed was calculated by measuring the time required for the millirobot to move a fixed distance of 5 cm.

### 2.6. Determination of In Vitro Drug Release

In vitro drug release from the hydrogel was evaluated using the dynamic dialysis method [36]. Hydrogel samples (5 mm diameter), either alone or fixed within the magnetic shell, were placed into dialysis bags (MWCO = 10,000 Da) containing PBS (pH 6.8). The volume ratio of the dialysis bag solution to that in the vial was 1:10. The vial was then placed in a constant-temperature water bath shaker at 37 °C and 150 rpm. At predetermined time intervals, 1 mL of the external release medium was collected and replaced with an equal volume of fresh PBS to maintain the stability of the release system. The collected samples were analyzed using a UV-Vis spectrophotometer (752N Plus, INESA, Shanghai, China) at 665 nm.

Drug release from the millirobot was evaluated under three pH conditions simulating the GIT (high, medium, and low) [37,38]. The medium pH condition corresponds to the normal GIT pH value, and the high and low pH conditions correspond to the abnormal GIT pH value. The pH values of the solution and the placement time are shown in Table 2, corresponding to simulated pH conditions and transit/drug-release times for the stomach, duodenum, lower small intestine, and colon. The millirobot was placed directly in a vial containing PBS, and the remaining methods were the same as those used to detect drug release from the hydrogel.

The cumulative drug release (*Q_n_*) at the nth sampling point can be calculated using the following equation:

(5) $$Q_{n}=\frac{C_{n}\times V_{0}+(C_{1}+C_{2}+\cdots \cdots +C_{n-1})\times V}{m_{0}}\times 100\%$$

In the equation, *V*_0_ is the total volume of the release medium (mL), *V* is the volume of the release medium collected each time (mL), *C_n_* is the drug concentration at the nth sampling point (µg/mL), and *m*_0_ is the initial amount of drug loaded in the sample (µg).

### 2.7. Evaluation of Unidirectional Drug Release from Millirobots

The unidirectional drug diffusion performance of the millirobots was characterized using a rectangular diffusion cell connected by a cylindrical channel (ID 10 mm). The millirobot was placed inside a silicone tube (OD 10 mm, ID 8 mm), which was then inserted into the cylindrical channel. This setup separated the drug-release side of the millirobot from the back side of the magnetic shell. Subsequently, 100 mL of PBS solution (pH 6.8) was injected into each compartment. To maintain sink conditions and ensure uniform drug concentration, the entire apparatus was placed on a dual-station thermostatic magnetic stirrer. At 2-h intervals, 1 mL samples were collected from the compartment on the back side of the shell to assess the unidirectional release performance of the millirobot.

## 3. Results and Discussion

### 3.1. Characterization of the Magnetic Shell

**Figure 3:**
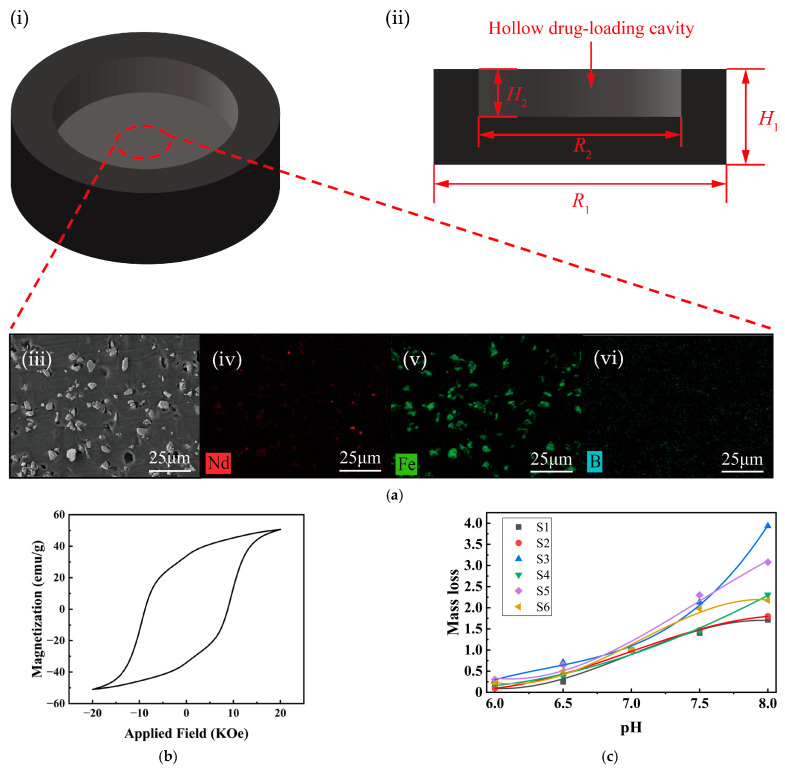
Characterization of the magnetic shell and pH-dependent film. (**a**) Schematic diagrams of the magnetic shell and corresponding EDS characterization: (i) isometric view showing the PDMS/NdFeB composite matrix; (ii) cross-sectional view with the hollow drug-loading cavity and dimensions; (iii) SEM image of the magnetic shell; (iv) Nd element map; (v) Fe element map; (vi) B element map. (**b**) Hysteresis loop of the magnetic shell. (**c**) Fitting curves of thin film dissolution rate.

To achieve biocompatibility and sufficient magnetic responsiveness for intestinal motion, the magnetic composite shell of the millirobot was fabricated using PDMS as the matrix and NdFeB magnetic powder as the magnetic functional phase, and its geometric configuration is illustrated in Figure 3(ai). As shown in Figure 3(aii), the hollow structure is designed for loading drug carriers. The structural dimensions are as follows: outer diameter *R*_1_ = 8 mm, inner diameter *R*_2_ = 5.5 mm, total height *H*_1_ = 2.6 mm, and the height of the hollow section *H*_2_ = 1.4 mm. As a flexible elastomer, PDMS can effectively reduce mechanical damage to surrounding tissues during the movement of the millirobot. Meanwhile, it offers low cost, simple fabrication, and good biocompatibility and is thus commonly used in the manufacture of millirobots for GIT drug delivery [39,40]. The magnetic shell adopts a tablet-like structure with smooth rounded edges, which is intended to reduce mechanical irritation during intestinal movement. The magnetic millirobot adopts a sheet-like structure with a low aspect ratio, which can reduce the minimum shear stress caused by constant peristalsis and mucosal fluid gradients, and prolong its intestinal retention time [41].

Energy Dispersive X-ray Spectroscopy (EDS) was used to characterize the magnetic shell via elemental mapping, as shown in Figure 3(aiii–vi). The characteristic elements of NdFeB magnetic powder, namely B, Fe, and Nd, were uniformly distributed without obvious large agglomeration. The signal points of the Nd element were widely and continuously distributed, with strong overlap with the Fe element distribution, indicating that the NdFeB magnetic powder was well dispersed in the PDMS matrix. Among them, the distribution of B element was relatively sparse, which may be attributed to two factors: first, B is a light element, and EDS has low detection sensitivity for it [42]; at the same time, a large number of magnetic powder particles were encapsulated inside the PDMS matrix, which further reduced the signal points of the B element. Figure S1 in the Supplementary Materials shows that the mass fraction of magnetic powder in the magnetic shell is about 18.7%, and the content of B element is about 4%, indicating that most of the magnetic powder is embedded within the PDMS matrix.

A Vibrating Sample Magnetometer (Model 8600 Series, Lake Shore, Westerville, OH, USA) was used to obtain the magnetization curve of the millirobot, and the results are shown in Figure 3b. Its saturation magnetization (*M_s_*) is about 50 emu/g, residual magnetization (*M_r_*) is about 33 emu/g, and coercivity (*H_c_*) is close to 9 kOe. This indicates that the magnetic millirobot can maintain a strong magnetic moment after magnetization and is difficult to demagnetize by weak external magnetic fields, ensuring magnetic stability during the magnetic drive process [43]. At the same time, the magnetization curve shows good linearity in low magnetic fields (1–5 kOe), making it suitable for precise magnetic control. In summary, the hysteresis loop demonstrates that the millirobot possesses a strong magnetic torque and excellent controllability, providing reliable performance guarantees for its magnetically driven movement in complex environments.

### 3.2. Formulation Selection of pH-Dependent Films

To realize site-specific drug release in the colon, the pH-dependent films were made from Eudragit L100 (EL 100) and Eudragit S100 (ES 100), both anionic acrylic resins containing carboxyl groups. In a low-pH environment, the carboxyl groups on the resin molecules exist as -COOH due to protonation, forming strong intermolecular forces that make the film water-insoluble, thereby effectively coating and protecting the drug. As the environmental pH increases, the carboxyl groups undergo deprotonation to form -COO^−^, and the molecular chains stretch due to electrostatic repulsion, forming soluble salts with cations in the medium, leading to gradual dissolution of the film and drug release. Due to its lower carboxyl group content, Eudragit S100 film has a higher dissolution pH threshold than Eudragit L100 film. In practical applications, EL 100 and ES 100 are often combined to flexibly adjust the dissolution pH range of the film [44]. When physiological conditions are abnormal, the pH-dependent film should not dissolve too quickly at high pH values or too slowly or not at all at low pH values. Therefore, it is necessary to determine the dissolution rate of the film under different pH values.

Given the high dissolution pH threshold of Eudragit S100 film, which would prevent film dissolution under low-pH conditions, Eudragit L100 film was selected as the primary film material. The plasticizer content of the Eudragit film not only alters the mechanical properties of the film but may also affect the dissolution properties of the film [45]. Therefore, six formulations S1–S6 (Table 1) were designed, and their mass loss under different pH conditions was measured as shown in Figure S2 in the Supplementary Materials.

To intuitively characterize the dissolution behavior of the film under different pH values, in this study, we performed normalization using the mass loss at pH = 7 as the reference (set to 1) and fitted the dissolution data with a cubic polynomial. The fitting coefficients and *R*^2^ are shown in Table A1 in Appendix A, with all *R*^2^ values being greater than 0.98, indicating an excellent fit; a more intuitive comparison is shown in Figure 3c. The mass loss at pH 6.1, 6.5, 6.8, 7.2, and 7.8 was calculated using the fitting equation. Assuming the film dissolution rate is constant at each time point, the dissolution rates for each sample under the three pH conditions (high, medium, and low) can be determined. Normalization processing was also performed to assess the dissolution quality of each sample within 3 h at a simulated small intestinal pH, and the results are shown in Table 3.

An ideal film should have the highest possible dissolution amount under low-pH release conditions and the lowest possible dissolution amount under high-pH release conditions; especially, the dissolution rate under low-pH conditions is more important. Based on these criteria, S1 and S4 are the most suitable. This is because their dissolved mass under low-pH conditions exceeds 70% of that under moderate-pH conditions, which is the highest among the six films. Meanwhile, under high-pH conditions, the dissolved mass of S1 is approximately 13% lower than that of S4. Finally, if the pH is only slightly above the pKa of the responsive polymer, the coating may dissolve slowly or even not at all [46], and the EL 100 film has the lowest dissolution threshold. Based on the above factors, S1 was selected as the pH-dependent film on the surface of the millirobot.

### 3.3. Characterization of the Drug Carriers

**Figure 4:**
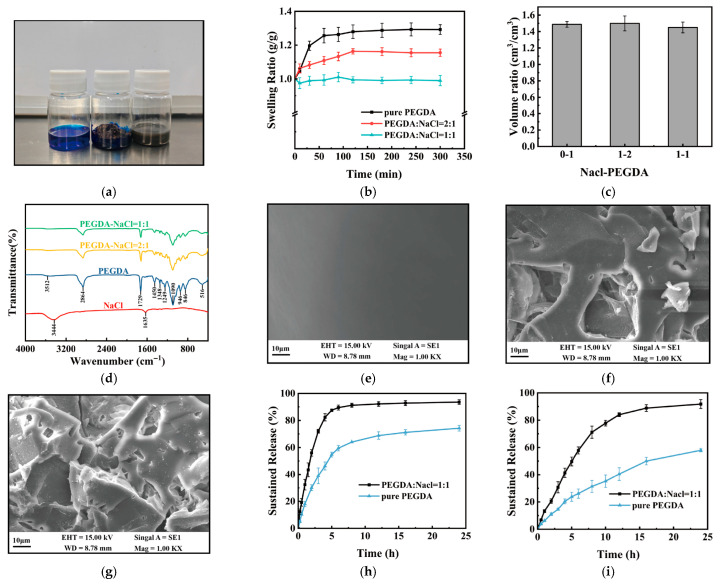
Characterization of drug carriers. (**a**) Photographs of the three drug carriers: deionized water (left), carbomer gel (middle), and PEGDA/NaCl hydrogel (right). (**b**) Swelling ratios of hydrogels with different PEGDA/NaCl ratios (n = 3; Mean ± SD). (**c**) The volume ratio of the hydrogels at equilibrium to their initial state (n = 3; Mean ± SD). (**d**) FT-IR spectra of NaCl powder, pure PEGDA, and PEGDA/NaCl hydrogel. (**e**–**g**) SEM images of hydrogels with different PEGDA: NaCl ratios: (**e**) 1:0, (**f**) 2:1, and (**g**) 1:1. (**h**) Drug release profiles of pure PEGDA and PEGDA/NaCl hydrogels without confinement, and (**i**) with confinement in the magnetic shell (n = 5; Mean ± SD).

Most miniature robots or targeted drug delivery systems can generally only carry a single type of drug carrier, such as a liquid [47] or a solid [48], which limits their versatility. In contrast, the design of the millirobot in this study, which uses PDMS adhesive to attach the pH-dependent film to the magnetic shell, enables the incorporation of various types of carriers. Deionized water, carbomer gel, and photocurable hydrogel are used to simulate three general types of drug carriers, demonstrating this versatility, as shown in Figure 4a. The model dye used is methylene blue, which is readily detectable via UV-Vis spectrophotometry and allows direct visual observation during release experiments.

Since the EL 100 film was used as the pH-dependent film, the drug carrier should not dissolve the film, especially in the liquid and gel states. Therefore, acetic acid was used to adjust the pH of the drug aqueous solution to approximately 3, and the carbomer gel was neutralized only to pH = 5, whereas the dissolution threshold of the EL 100 film is 6 [49]. Because the aqueous solution is incompressible, filling the interior with it may cause liquid to leak during film installation. Therefore, only 20 μL of the drug-loaded aqueous solution was added.

For solid drugs, such as hydrogels or tablets, volume changes (e.g., swelling or disintegration) occur during drug release. This will lead to the failure of unidirectional drug release of the millirobot. To address this, a chemically crosslinked photocurable hydrogel with a low swelling rate was used, and the size of the hydrogel was reduced. In addition, a ring-shaped EC film attached to the shell was used for restriction. PEGDA was selected as the photocurable hydrogel, which is commonly used in millirobots for its good biocompatibility, ease of molding, and controllable structure [50].

However, a low volume change rate may result in a slow drug release rate. NaCl was used as a channeling agent to enhance the drug-release rate [51], and two hydrogel formulations with PEGDA: NaCl mass ratios of 2:1 and 1:1 were prepared. The incorporation of a large amount of the channeling agent can change the swelling and drug release properties of the hydrogel. The swelling rates of hydrogels with different ratios were measured, and the results are shown in Figure 4b. The swollen mass of hydrogels containing NaCl was much smaller, and the mass of PEGDA/NaCl = 1:1 was almost unchanged. Interestingly, as shown in Figure 4c, the maximum swelling volume differed slightly. This may be because, although NaCl does not directly participate in hydrogel crosslinking, it stretches the network during the crosslinking process to form a fixed structure, while NaCl is continuously lost.

FT-IR spectroscopy analysis of the hydrogels also confirmed this point. As shown in Figure 4d, the absorption peak positions of PEGDA and PEGDA/NaCl hydrogels were the same, and only the intensity changed, indicating that the addition of NaCl did not change the chemical structure of PEGDA, but only changed the microstructure of PEGDA through physical occupancy/phase separation [52]. The scanning electron microscopy (SEM) images of the hydrogels are shown in Figure 4e–g. Compared with pure PEGDA hydrogels, PEGDA/NaCl hydrogels exhibit a rougher microstructure with numerous interconnected pores, indicating that the addition of NaCl increases pore size and significantly modifies their network structure.

The mass loss of PEGDA/NaCl = 1:1 hydrogel after swelling in water for a specific time and drying was measured, and the results are shown in Figure S3 in the Supplementary Materials, indicating that a large amount of NaCl in the hydrogel was lost over time; a more intuitive comparison is shown in Figure S4 in the Supplementary Materials. Meanwhile, the swelling rate of the PEGDA/NaCl hydrogel when re-swollen after drying at the equilibrium swelling rate was measured, and the results are shown in Figure S5 in the Supplementary Materials, which is much higher than that of the pure PEGDA hydrogel. This indicates that although a large amount of NaCl has been lost, the chemically crosslinked network has been changed.

The drug release rates of different hydrogels are shown in Figure 4h. The hydrogel with added NaCl showed faster, more complete drug release, which was more pronounced when the hydrogel was placed inside the magnetic shell. As shown in Figure 4i, PEGDA released less than 60% of the drug within 24 h, while the PEGDA/NaCl hydrogel released more than 90% of the drug. This may be because although NaCl has been lost, PEGDA/NaCl has larger pores, lower crosslinking density, and a looser network. In summary, the PEGDA/NaCl hydrogel has a slightly lower maximum swelling volume than pure PEGDA and a faster drug release rate, making it a more suitable solid drug carrier model.

### 3.4. In Vitro Drug Release of the Millirobot

**Figure 5:**
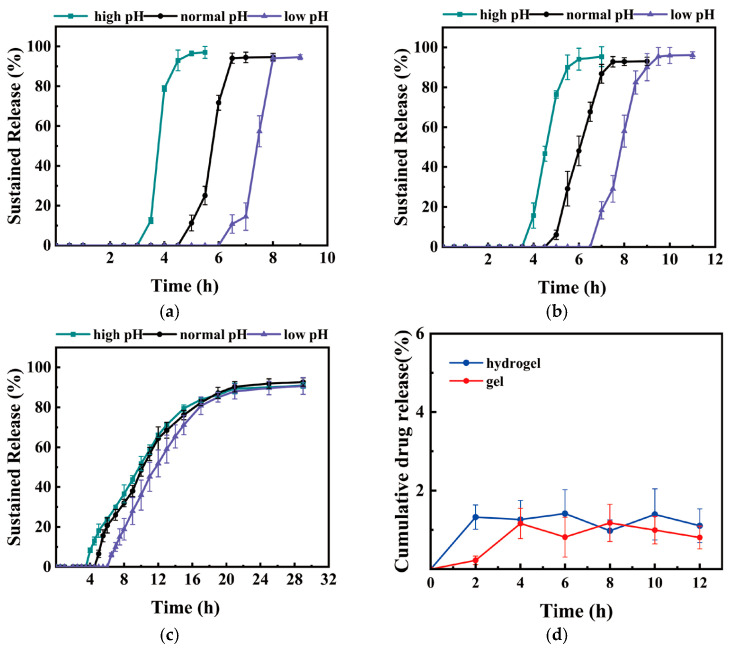
Drug release of the millirobot in simulated in vitro environments. (**a**) Drug release profile of the millirobot loaded with the aqueous solution (n = 5; Mean ± SD). (**b**) Drug release profile of the millirobot loaded with gel (n = 5; Mean ± SD). (**c**) Drug release profile of the millirobot loaded with hydrogel. (**d**) Unidirectional drug release profiles of gel and hydrogel (n = 5; Mean ± SD).

The in vitro drug release of the millirobot was measured under three different simulated GIT conditions, as described in Section 2.6. The drug release rates of the aqueous solution, carbomer gel, and PEGDA/NaCl hydrogel are shown in Figure 5a–c. As the film thickness increases, the onset time of drug release is prolonged, and adjusting the film thickness can directly affect the drug release of the millirobot [53]. The 110 μm-thick S1 film can only ensure no drug leakage for 3.5 h in the moderate-pH environment (including 2 h in an acidic environment), so the 180 μm-thick S1 film was finally selected. The S1 films prepared in different batches show stable performance, proving that the batch has little influence on the film.

Under medium-pH conditions, the drug release from all three carriers began after a lag time of approximately 4.5–5 h, corresponding to the distal small intestine to the colon. At this time, only the central part of the pH-dependent film had dissolved; therefore, even the aqueous solution released only a small portion of the drug. As the film gradually dissolved, the drug release began to accelerate. The aqueous solution released approximately 46% of the drug within 5.5 to 6.5 h. As the film dissolution approached the film edge, the film dissolution rate began to decrease; meanwhile, the bottom material exerted a tensile force on the aqueous solution, further decreasing in the drug release rate. The drug was almost completely released after approximately 2 h of release, whereas the aqueous solution without the film released almost instantly upon contact with water. The gradual dissolution of the film also affected the drug-release rates of the carbomer gel and PEGDA/NaCl hydrogel. For example, the hydrogel without the film released approximately 70% of the drug within 10 h, whereas the hydrogel with the film required 13 h.

Under the simulated high and low pH conditions, drug release started at 6.5 or 7 h under the low pH simulation, which was 1.5 to 2 h later than the 5 h in the simulated stomach and small intestine. In contrast, under the high-pH simulation, drug release began at 3.5 to 4 h, and the film dissolved for 1.5 to 2 h under the simulated small-intestinal pH environment before drug release occurred. After the film began to dissolve, the release rates of the three drug carriers were similar to those observed under the medium-simulated pH condition, especially for hydrogel drug release, where the curves almost overlapped after a period of release.

The drug release behaviors of both PEGDA hydrogel-based millirobots and carbomer gel-loaded millirobots were kinetically fitted using four classical models: zero-order, first-order, Higuchi, and Korsmeyer–Peppas (KP). The detailed fitting parameters are summarized in Tables S1 and S2 in the Supplementary Materials.

It was observed that both gel systems showed good correlation with the KP model. However, for the carbomer gel-loaded millirobots, the KP model yielded release exponents *n* > 1 under all pH conditions, which corresponds to a release profile with increasing release rate over time. This result does not conform to the physical assumptions of the classical single-stage power-law model, and can be attributed to the biphasic release behavior induced by the outer pH-dependent film: an initial lag phase due to film sealing, followed by accelerated drug release once the film dissolves to a critical extent. Consistent with this mechanism, the sudden and massive release of the model dye (methylene blue) was observed during the release experiments.

Furthermore, both the hydrogel and gel systems showed very high correlation with the first-order release model, indicating that diffusion remained the dominant release mechanism for both systems. This apparent contradiction with the KP model results may arise from the different applicable scopes of the two models: the former targets the first 60% of cumulative release for fitting, while the latter fits the entire release process. This discrepancy in turn proves the influence of the film on the drug release behavior, and further analysis of the KP model exponents also reveals that film dissolution significantly regulates the early-stage release behavior. For the PEGDA hydrogel-based millirobots under low pH conditions, the *n* value exceeded 0.85, suggesting that the slow dissolution of the pH-dependent film restricted the swelling of the inner PEGDA hydrogel. Once the film dissolved sufficiently, the concentration gradient of the drug became the dominant driving force for release. Considering that only the hydrogel-based millirobots under medium and high pH conditions exhibited *n* values < 0.85, it is likely that the degree of film influence is negatively correlated with the ratio of the film dissolution rate to the drug release rate.

The NaCl-containing hydrogel exhibited lower *n* values in the KP model, indicating that diffusion plays a more dominant role than in the NaCl-free hydrogel. However, when encapsulated within the millirobot shell, the contribution of diffusion decreases due to the influence of the pH-dependent film. As mentioned earlier, the degree of film influence is governed by the ratio of the film dissolution rate to the drug release rate. Thus, the contributions of film dissolution, hydrogel structure, and diffusion vary with pH conditions and drug carriers during the early stage of millirobot release, while diffusion becomes the dominant driving force in the middle and late stages of drug release.

Both the zero-order and Higuchi models showed poor correlation with the release profiles of all millirobot systems. Interestingly, there is a significant difference in model fitting correlation between NaCl-containing and NaCl-free PEGDA hydrogels. This may be attributed to the fact that the structure of PEGDA/NaCl hydrogel is closer to a rigid porous matrix compared to pure PEGDA hydrogel after the addition of NaCl.

Only one side of the magnetic shell of the millirobot can release drugs, while the other impermeable PDMS/NdFeB side restricts the direction of drug diffusion. This helps reduce drug diffusion into the intestinal lumen, thereby improving drug absorption efficiency [54]. A diffusion cell was used to determine the sustained-release curve of the model dye from the backing layer (the back side of the millirobot). The results are shown in Figure 5d, which shows that less than 2% of the drug was released in this direction. A more intuitive comparison is provided in Figure S6 of the Supplementary Materials, where Figure S6a shows the release of carbomer gel and Figure S6b shows the release of PEGDA/NaCl hydrogel.

In summary, the millirobot demonstrated pH-dependent release behavior consistent with its design objectives. The drug can be released within a reasonable time under conditions that simulate normal physiological conditions. The drug can be released within a relatively short time under low-pH conditions, and maintain sufficient time to protect the drugs under high-pH conditions. This provides the possibility of in vitro magnetic field intervention in abnormal physiological environments.

### 3.5. Motion of the Millirobot

**Figure 6:**
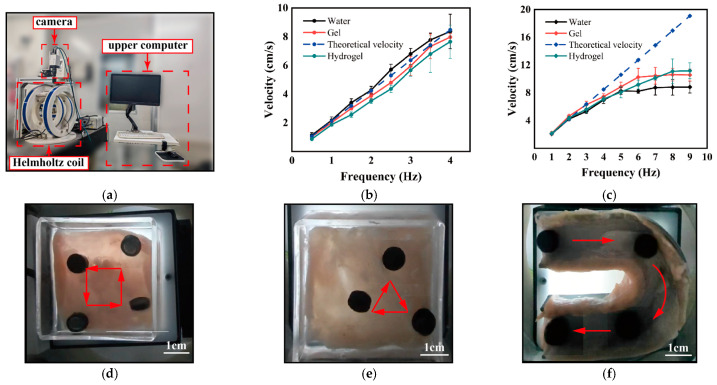
Millirobot movement. (**a**) Three-dimensional Helmholtz coil. (**b**) Movement speed of the millirobot in the air environment (n = 5; Mean ± SD). (**c**) Movement speed of the millirobot in deionized water (n = 5; Mean ± SD). (**d**) A millirobot loaded with hydrogel is moving along a rectangular trajectory in an environment containing a small amount of liquid. (**e**) A millirobot loaded with an aqueous solution moves along a triangular trajectory in a deionized water environment. (**f**) A millirobot loaded with gel tumbling through a U-shaped track in the air environment. The red arrows in (**d**–**f**) indicate the movement direction of the millirobot.

As shown in Figure 6a, the millirobot was driven by a three-dimensional Helmholtz coil, which generated a uniform magnetic field in the central region by precisely controlling the current. The millirobot moves in a tumbling mode under the action of the rotating magnetic field. When the external magnetic field rotates continuously, the millirobot is subjected to a magnetic torque to overcome gravity, electrostatic forces, and adhesion, thereby generating a tumbling motion. When there is an asymmetric interaction between the robot and the contact surface such as the intestinal wall, the tumbling motion is converted into net forward locomotion [55]. According to classical electromagnetic theory, the magnetic torque *T*_m_ (N·m) of the millirobot in the magnetic field can be calculated using Equation (6):

(6) ***T*****_m_** = *V* (**M** × **B**)

where *V* is the volume of the magnet (m^3^), **M** is the magnetization intensity of the magnet (A m^−1^), and **B** is the magnetic flux density at the position of the magnet (T).

The EL 100 film provides the time required for the millirobot to move, and the movement speed of the millirobot is equally important for in vitro intervention. The movement speed of the millirobot is greatly affected by the magnetic field frequency. Therefore, the relationship between the movement speed of the millirobot and the magnetic field frequency was measured in air and liquid environments, with the magnetic flux density held constant at 8 mT. Since the millirobots loaded with different drug carriers have different masses, the movement speeds of the three types of millirobots were measured separately. The results are shown in Figure 6b,c, and all speed measurements were performed on the surface of the ex vivo intestinal tract.

The theoretical speed of the millirobot at frequency *f* is 2*f* × (*R* + *h*), where *R* is the OD of the millirobot, *f* is the magnetic field frequency and *h* is the thickness of the millirobot. This rough calculation method is relatively accurate in low-frequency air environments. As shown in Figure 6b, the experimental and theoretical moving velocities of the millirobots increased linearly at a similar rate within the range of 0.5–4 Hz. Among them, the magnetic millirobot loaded with water exhibited the smallest deviation, with a maximum deviation of only approximately 7.8% at 4 Hz. At this point, their moving velocity reached about 8.4 cm/s, which is 10 times their body length. This is because the magnetic millirobot loaded with water has the smallest mass, moment of inertia, and resistance. The millirobot loaded with Carbomer gel shows moderate deviations, with a maximum deviation of approximately 9.3%. The millirobot loaded with hydrogel, due to their maximum mass, possessed the largest moment of inertia, as well as the highest adhesion forces. This made it more difficult for them to follow the rotating frequency of the magnetic field, resulting in the largest deviation from the theoretical velocity. Specifically, their deviation reached up to 19.6% at 0.5 Hz, and the minimum deviation was still 8.4% at 3.5 Hz. This may be because the mass difference between the Carbomer gel and the aqueous solution is small. By contrast, the hydrogel contains a large amount of NaCl, whose density is approximately twice that of water, thus giving it a much higher overall mass. Notably, as the frequency increases, the deviation of the millirobot loaded with hydrogel from the theoretical speed decreases. This may be attributed to its inability to quickly overcome the moment of inertia at low frequencies. At 4 Hz, the maximum speed is about 7.7 cm/s, only about 8.3% slower than the millirobot loaded with water. When the frequency exceeds 4 Hz, the millirobot cannot fully contact the bottom surface during tumbling and may “jump”, thereby affecting movement stability.

In water, the motion speed of the millirobot increases linearly with frequency in the range of 1–4 Hz, and the deviation from the theoretical speed is generally less than 20%. However, when the frequency exceeded 4 Hz, the millirobots showed large deviations from the theoretical velocity and gradually no longer maintained a linear growth. The buoyancy of water reduces the contact between the millirobot and the bottom surface. Since tumbling locomotion relies on asymmetric friction to generate forward motion, reduced friction not only lowers the ability of the millirobot to overcome drag but also causes slip. Meanwhile, the viscous drag of water increased significantly with velocity, further reducing the moving velocity of the millirobots. These factors ultimately led the experimental velocity to enter a saturation plateau, and the deviation from the theoretical model continued to expand. The millirobot loaded with hydrogel, having the largest mass and the largest normal force, maintains tighter contact with the intestinal surface, exhibiting the best anti-slip performance, the highest cut-off frequency (8 Hz), the highest saturation speed (11.1 cm/s), and the smallest deviation from the theoretical model at high frequencies. In contrast, the millirobot loaded with water, most affected by buoyancy, reaches saturation at 5 Hz, with a saturation speed of about 8.8 cm/s and the largest deviation at high frequencies.

The speed of millirobot is measured on different porcine colon slices. The relative standard deviation (RSD) of speed was below 10% at most conditions. The maximum RSD of approximately 18.7% observed for the millirobot (loaded with hydrogel) in air environment at 3.5 Hz, indicating good stability on the ex vivo porcine colon surface.

It is also important that the millirobot can respond quickly to changes in magnetic torque direction. The millirobot containing hydrogel has the largest mass and thus needs to overcome the greatest resistance. In the intestinal environment with only a small amount of PBS solution, the millirobot can move along a rectangular trajectory, as shown in Figure 6d (see Video S1 in Supporting Information). The millirobot containing aqueous solution has the smallest mass and can move along a triangular trajectory in an environment with plenty of PBS solution, as shown in Figure 6e (see Video S2 in Supporting Information). A U-shaped track was used to simulate the intestinal bending segment. As shown in Figure 6f (see Video S3 in Supporting Information), the magnetic millirobot loaded with carbomer gel can successfully pass through the U-shaped track, indicating that the millirobot has good controllability.

In summary, although the movement speeds of millirobots loaded with different drug carriers are slightly different, the controllable speed in air is about 8 cm/s, and the maximum speed in water exceeds 10 cm/s, showing a relatively fast movement speed. Meanwhile, the ability to follow a specific trajectory further demonstrates the good controllability of the millirobot, which provides the possibility for in vitro intervention under abnormal physiological conditions.

## 4. Conclusions

In this study, a colon-targeted millirobot with rational material design, integrating a pH-dependent strategy and magnetic actuation was successfully developed, which can carry various types of drug carriers.

The magnetic shell of the millirobot was fabricated using PDMS and NdFeB magnetic powder for biocompatibility and magnetic responsiveness. EDS elemental mapping and VSM magnetization curves confirmed that the NdFeB magnetic powder was uniformly dispersed in the PDMS matrix without obvious agglomeration. The magnetic shell exhibited a remanence of approximately 33 emu/g and a coercivity of nearly 9 kOe. Driven by an 8 mT rotating magnetic field, the millirobot achieved stable tumbling motion with controllable speeds of up to 8 cm/s in air and 10 cm/s in water, and could move along rectangular, triangular, and U-shaped trajectories. The favorable motion speed and controllability ensure the feasibility of in vitro intervention under abnormal physiological conditions.

The pH-dependent film was prepared from Eudragit L100, which exhibited appropriate delayed drug release behavior in different pH environments. Under simulated normal intestinal physiological conditions in vitro, the millirobot released the drug within 2.5–3 h at a medium pH, corresponding to the distal small intestine to the colon. Under simulated abnormal physiological conditions, drug release was delayed by 1.5–2 h in a low-pH environment, and the drug could be protected for 1.5–2 h even in a high-pH environment, reserving sufficient operation time for external magnetic intervention.

The millirobot shows high versatility and can carry different drug carriers. In this study, deionized water (liquid), carbomer gel (semi-solid), and PEGDA/NaCl hydrogel (solid) were used to assess adaptability to various carriers of the millirobot. Among them, unidirectional drug release from solid carriers is the most challenging—carriers with low volume change tend to show slow release, while those with high volume change may detach from the shell. This issue was solved by adding a porogen into the PEGDA hydrogel. The drug release rate of PEGDA/NaCl within the magnetic shell exceeded 90% at 24 h, achieving a combination of low volume change and fast release. The three drug carriers exhibited fast, medium, and slow-release rates, respectively.

In vitro simulated drug-release experiments demonstrated that the millirobot could achieve targeted release using the pH-dependent film without magnetic intervention under normal physiological conditions. Under abnormal physiological conditions, the millirobot could be manipulated to reach the target region via an external magnetic field. In addition, the unidirectional release design based on the magnetic shell reduced drug leakage from the backing layer to less than 2%, effectively enhancing intestinal drug absorption efficiency.

In summary, the magnetically actuated pH-dependent millirobot developed in this study provides a new strategy for colon targeting. By effectively integrating traditional pH-dependent targeting and magnetic actuation, it is expected to address the limitations of conventional colon-targeted delivery systems, which are susceptible to abnormal physiology, as well as the high cost of single magnetic carriers. Meanwhile, the designed millirobot exhibits advantages including high carrier versatility and unidirectional drug release. The soft PDMS material, together with the tablet-like geometry inspired by oral tablets, is intended to improve the safety of the millirobot. The motion performance of the millirobot was tested in an ex vivo porcine intestine, and its drug release performance was tested in a simulated gastrointestinal pH environment in vitro, which achieved desirable locomotion speed and drug release behavior. However, the static intestinal model used cannot replicate the complex physiological environment of the real gastrointestinal tract, and the simulated pH conditions also deviate from the actual in vivo situation. Therefore, comprehensive in vivo experiments are still required to further evaluate the biosafety, motion performance, and drug release performance of the millirobot. This will be the focus of our future work.

## Figures and Tables

**Table 1:** Composition of Eudragit L/S films.

Formulation	Eudragit L 100 (g)	Eudragit S 100 (g)	Triethyl Citrate (g)
S1	10	0	2
S2	10	0	4
S3	8	2	2
S4	8	2	4
S5	5	5	2
S6	5	5	4

**Table 2:** pH values and corresponding times for three different simulated conditions.

pH Condition	2 h	1 h	2 h	Until End
High	1.2	7.2	7.8	7.8
Medium	1.2	6.5	7.2	6.8
Low	1.2	6.4	7	6.5

**Table 3:** Mass loss of EL/ES 100 films.

Formulation	Low-pH Condition (g)	Medium-pH Condition (g)	High-pH Condition (g)
S1	0.7116	1	1.5282
S2	0.6841	1	1.4768
S3	0.6464	1	2.0438
S4	0.7161	1	1.7562
S5	0.5756	1	1.5493
S6	0.5913	1	1.6116

## Data Availability

The dataset is available upon request from the authors.

## Supplementary Materials

The following supporting information can be downloaded at https://www.mdpi.com/article/10.3390/mi17050610/s1 .

## Appendix A. Fitting of Mass Loss for the pH-Dependent Film

A trinomial equation, *y* = *ax*^3^ + *bx*^2^ + *cx* + *d*, was employed to fit the mass loss data, where *y* represents mass loss and *x* represents pH value. The coefficients and the coefficient of determination of fit *R*^2^ are shown in Table A1.

**Table A1:** Quadratic ternary fitting coefficients and coefficient of determination.

	*a*	*b*	*c*	*d*	*R* ^2^
S1	−0.468509	9.836919	−67.574356	152.606639	0.990
S2	−0.276607	5.785714	−39.213042	86.835927	0.999
S3	0.543427	−10.385815	66.800763	−143.997410	0.998
S4	−0.027778	0.913095	−7.605556	18.919286	0.993
S5	−0.348653	7.839971	−56.770665	134.030242	0.985
S6	−0.656009	13.858850	−95.953645	218.734179	0.989
