# Supplementary material for: Development of pH-Dependent Magnetically Actuated Millirobot for Colon-Targeted Delivery of Diverse Drug Types
Source: Micromachines (Basel). 2026 May 15;17(5):610. doi: 10.3390/mi17050610 (PMC13209673; doi:10.3390/mi17050610)
Supplement: Supplementary file 1 [file micromachines-17-00610-s001.zip › micromachines-4262084-supplementary.pdf]

Supplementary Materials

Hydrogel mass loss

The weight of the hydrogel was measured and recorded as m_1, and then the hydrogels were placed in PBS solution. Samples were taken out at predetermined time intervals and dried in a constant-temperature drying oven at 50 °C. After complete drying, the weight of the samples was measured and recorded as m_2. The mass loss rate of the hydrogel in the solution can be calculated using the following formula:

$$R_1 = \frac{m_1 - m_2}{m_2} \tag{S1}$$

Hydrogel reswelling rate and mass ratio

Sheet-shaped PEGDA/NaCl hydrogels were prepared and their initial weight was recorded as m_3. After swelling for 2 hours, the hydrogels were thoroughly dried in a vacuum drying oven and re-weighed, with this weight recorded as m_4. Then, the dried hydrogels were immersed in a PBS water bath at 37 °C, taken out at specific time intervals, wiped, and weighed, with the weight of the hydrogel at this time recorded as m_5. The reswelling rate of the hydrogel after re-swelling was calculated by the following formula:

$$R_2 = \frac{m_5 - m_4}{m_4} \tag{S2}$$

The mass ratio of the hydrogel after reswelling to its initial state was calculated by the following formula:

$$R_3 = \frac{m_5}{m_3} \tag{S3}$$

The results are shown in Figure S5.

Hydrogel microstructure

The cross-sectional morphology of the hydrogels was observed using a scanning electron microscope (Sigma 300, Zeiss) operated at an accelerating voltage of 10 kV. Before SEM characterization, the hydrogels were rapidly frozen in liquid nitrogen to form amorphous ice, and then fractured with a blade. The fractured samples were freeze-dried in a freeze dryer (SCIENTZ-10N, Xinzhi Biotechnology) at -45 °C for 24 hours.

Drug Release Kinetics Fitting Results

Four classical drug release kinetic models were employed to quantitatively analyze the in vitro drug release behavior of hydrogel carriers and millirobots, including the zero-order model, first-order model, Higuchi model, and Korsmeyer-Peppas (KP) model. Their mathematical expressions are shown in Equations (S4)–(S7):

$$Q_t = k_0 t + Q_0 \tag{S4}$$

In the above formula, Q_t (%) refers to the cumulative drug release percentage at time t; k_0 (% · h^{-1}) is the zero-order release rate constant; Q_0 (%) represents the initial drug release amount.

$$Q_t = Q_\infty (1 - e^{-k_1 t}) \quad \text{(S5)}$$

In the above formula, Q_∞ (%) is the maximum cumulative drug release percentage; k_1 (h^{-1}) denotes the first-order release rate constant.

$$Q_t = k_H \sqrt{t} + b \quad \text{(S6)}$$

In the above formula, k_H (% · $h^{-1/2}$) is the Higuchi release rate constant; b (%) is the intercept of the Higuchi model.

$$Q_t = k_p t^n \quad \text{(S7)}$$

In the above formula, k_p is the rate constant of the KP model; n is the release exponent, which is used to distinguish the mass transport mechanism of drug release.

For the tablet-like drug carriers, the critical range and mechanism classification of the KP release exponent n are defined as follows:

$n \approx 0.43$: Pure Fickian diffusion. $0.43 < n < 0.85$: Anomalous non-Fickian diffusion. $n \approx 0.85$: Pure Case-II transport. $n > 0.85$: Super Case-II transport.

For the PEGDA hydrogel drug carriers, the free release profiles of bare hydrogels and the drug release profiles of hydrogels encapsulated within the millirobot shell were fitted separately. The obtained kinetic parameters are summarized in Table S1, where "Free hydrogel" refers to the bare hydrogel control group without shell encapsulation.

Table S1. Drug release kinetic parameters of PEGDA hydrogel-based systems under different conditions.

Model	Parameter	Free hydrogel (without NaCl)	Free hydrogel (with NaCl)	Millirobot (high pH)	Millirobot (medium pH)	Millirobot (low pH)
Zero-order	k_0	3.0338	3.3956	3.8447	4.0140	4.3761
	Q_0	22.5632	41.5586	18.9930	19.6142	16.5007
	R^2	0.6731	0.4898	0.8416	0.8443	0.8432
First-order	k_1	0.2957	0.4618	0.1184	0.1273	0.1233
	Q_∞	72.9911	94.1800	100.4307	100.7494	101.7002
	R^2	0.9985	0.9967	0.9899	0.9923	0.9916
Higuchi	K_H	17.5146	39.3354	21.4973	22.4886	23.6801
	b	4.9541	-4.5472	-6.2581	-4.3282	-9.4988
	R^2	0.8983	0.9652	0.9594	0.9597	0.9404
KP	k_p	32.7561	18.0404	11.9458	13.2876	10.4079
	n	0.7568	0.6799	0.7767	0.7725	0.8970
	R^2	0.9988	0.9974	0.9948	0.9875	0.9994

The same kinetic analysis method was applied to fit the drug release profiles of millirobots loaded with carbomer gel, and the obtained kinetic parameters are summarized in Table S2.

Table S2. Drug release kinetic parameters of millirobots loaded with gel under different pH conditions.

Model	Parameter	Millirobot (high pH)	Millirobot (medium pH)	Millirobot (low pH)
Zero-order	k_0	29.9415	24.2283	23.6825
	Q_0	12.1710	7.6026	14.1732
	R^2	0.8378	0.8735	0.8402
First-order	k_1	0.4586	0.5810	0.3352
	Q_∞	120.3530	118.5792	132.6737
	R^2	0.9519	0.9538	0.9461
Higuchi	K_H	58.3418	46.5190	42.1358
	b	-8.0553	-10.2224	-4.5108
	R^2	0.8395	0.8196	0.8518
KP	k_p	44.7031	26.8225	33.8100
	n	1.3461	1.3632	1.2577
	R^2	0.9976	0.99124	0.9757

Figure

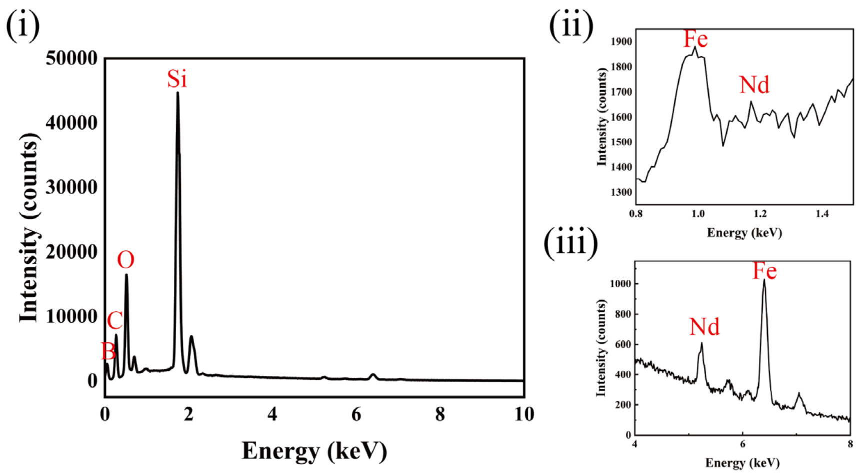

Figure S1. Total EDS spectrum of the PDMS/NdFeB shell of the magnetic millirobot. (i) Full-range EDS spectrum showing the characteristic peaks of elements from the PDMS matrix (B, C, O, Si). (ii) Magnified view of the low-energy region (0.8 – 1.4 keV), highlighting the low-energy characteristic peaks of Fe and Nd. (iii) Magnified view of the high-energy region (4 – 8 keV), showing the standard main characteristic peaks of Nd (Lα) and Fe (Kα). EDS quantitative analysis shows the weight percentages of elements: Si (37.5%), C (26.2%), O (15.3%), Fe (9.7%), Nd (7.0%), and B (4.3%).

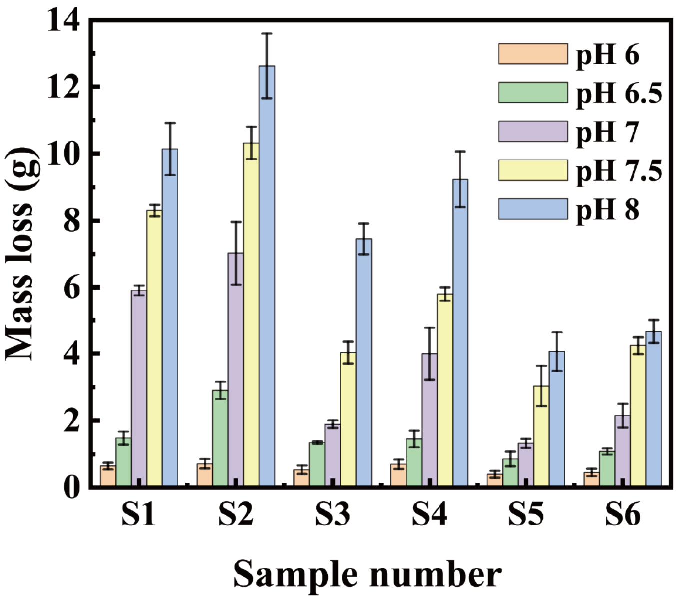

Figure S2. Mass loss of Eudragit L/S 100 films of samples 1 to 6 at different pH values. (n = 5; Mean ± SD).

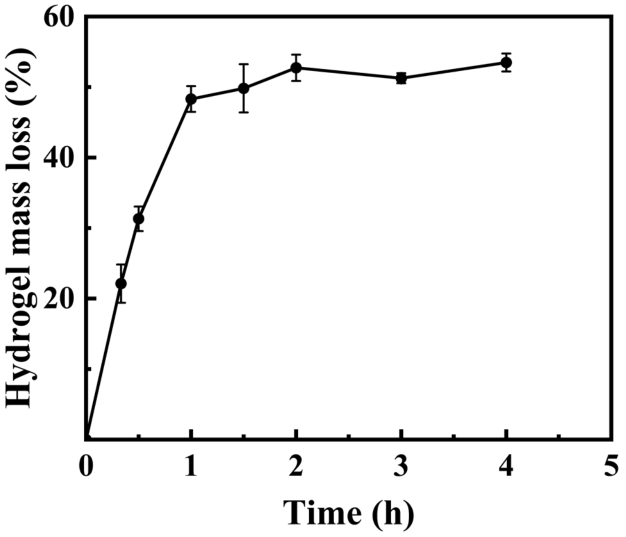

Figure S3. Mass loss of PEGDA/NaCl = 1:1 hydrogel.

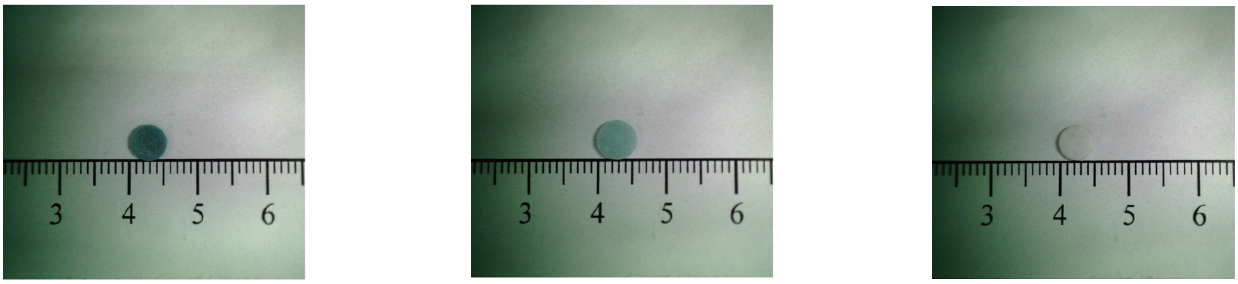

(**a**) (**b**) (**c**)

Figure S4. Images of PEGDA/NaCl = 1:1 hydrogel under various states. (a) Initial state. (b) After reaching swelling equilibrium. (c) After drying.

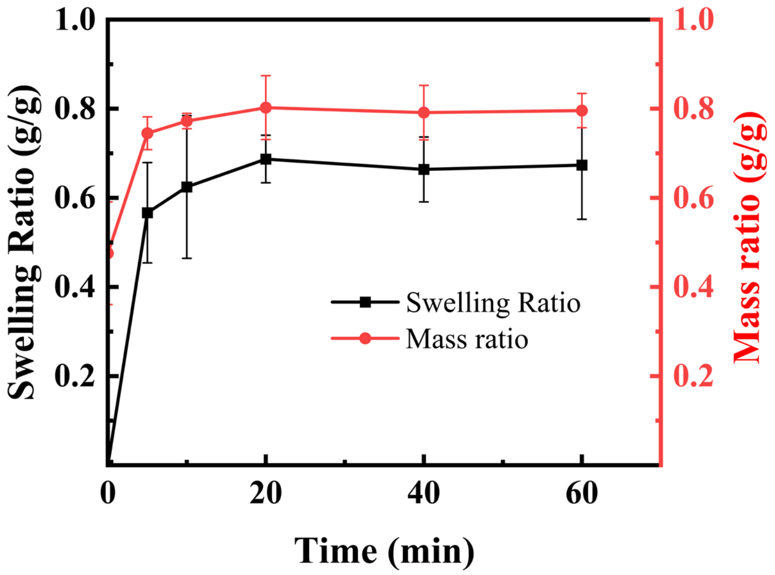

Figure S5. Reswelling rate and mass ratio of PEGDA/NaCl = 1:1 hydrogel.

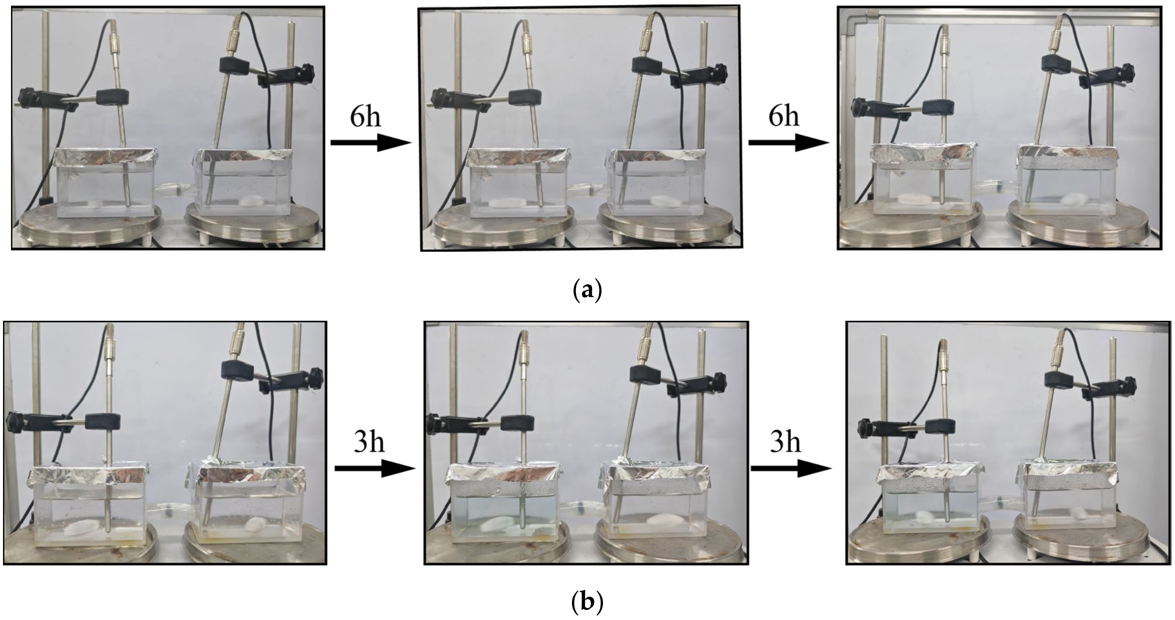

Figure S6. Millirobot unidirectionally releases drug image in customized diffusion cell. (a) Release image of a millirobot loaded with hydrogel. (b) Release image of the millirobot with gel.
